# Safety profile of robotic-assisted transperineal MRI-US-fusion guided biopsy of the prostate

**DOI:** 10.3389/fonc.2022.1025355

**Published:** 2022-12-01

**Authors:** Manuel Walter, Pawel Trotsenko, Hanns-Christian Breit, Nicola Keller, Anja Meyer, David Jean Winkel, Hans Helge Seifert, Christian Wetterauer

**Affiliations:** ^1^ Department of Urology, University Hospital Basel, Basel, Switzerland; ^2^ Department of Radiology, University Hospital Basel, Basel, Switzerland; ^3^ University of Basel, Basel, Switzerland; ^4^ Department of Medicine, Faculty of Medicine and Dentistry, Danube Private University, Krems, Austria

**Keywords:** biopsy, prostate, robotic-assisted, safety, transperineal

## Abstract

**Introduction:**

Robotic-assisted transperineal MRI-US-fusion guided biopsy of the prostate is a novel and highly accurate procedure. The aim of this study was to evaluate the MonaLisa prostate biopsy system in terms of safety, tolerability, and patient-related outcomes.

**Methods:**

This prospective study included 228 patients, who had undergone Robotic-assisted transperineal MRI-US-fusion guided biopsy of the prostate at the University Hospital Basel between January 2020 and June 2022. Peri-operative side effects, functional outcomes and patient satisfaction were assessed.

**Results:**

Mean pain score on the day of biopsy was 1.3 points on VAS, which remained constant on the day after biopsy. Overall, 32 of 228 patients (14%) developed grade I complications according to Clavien-Dindo classification. No higher-grade complications occurred. Gross haematuria, hematospermia and acute urinary retention occurred in 145/228 (63.6%), 98/228 (43%) and 32/228 (14%) patients, respectively. One patient (0.4%) developed urinary tract infection.

**Conclusions:**

Robotic-assisted transperineal MRI-US-fusion guided biopsy of the prostate performed under general anesthesia is a safe and well tolerated procedure. This technique allows to omit perioperative prophylaxis and at the same time minimizes the risk of infectious complications. We attribute the favorable risk profile and tolerability to the minimal invasive approach *via* two entry points.

## Introduction

Prostate cancer (PCa) is the second most common malignant disease in men worldwide (1) Suspicion for PCa is based on pathological digital rectal examination (DRE), prostate specific antigen (PSA) or magnetic resonance image (MRI) findings and indicates, as standard of care, a biopsy of the prostate (PB_x_) for histopathological verification (2). PB_x_ represents one of the most common urological procedures, with more than 1 million interventions performed in Europe and the United States every year (3). PB_x_ can be performed *via* a transrectal (TR) or transperineal (TP) route, each approach being associated with specific benefits and limitations. TR offers practicability in the in-office setup due to feasibility under local anesthesia reflected by the majority of PB_x_ being performed *via* the TR approach in the US (93.1 – 99.2%) (4). However, punction of the prostate through the rectum ampulla is associated with a significant risk for infectious complications (5). The incidence for infectious complications after TR-PB_x_ ranges between 5 and 7% with a hospitalization rate of about 2% (2, 3). Rising rates of fluorchinolone-resistance organisms, which could be found in up to 30% of rectal swab cultures prior to TR-PB_x_, possibly aggravate the situation (2). With the TP approach infectious complications are significantly lower, even negligible (2, 6, 7). Technological advances in diagnostics of PCa, like the implementation of multiparametric MRI (mpMRI) and MRI-targeted PB_x_ have increased the detection rate of significant PCa, simultaneously decreasing the detection rate of clinical insignificant PCa (8). Newly available robotic-assisted biopsy systems like MonaLisa combine the robotic precision with the preferable transperineal approach. Furthermore, this system allows for a minimal-invasive and gentle sampling requiring only two puncture sites and thus promising lower complication rates and patient tolerability. The robotic-assisted MRI-TRUS-fusion allows for highly precise biopsies with maximal reproducibility, while safely sparing the neurovascular bundle. So far there are no prospective reports on patient related outcomes in terms of tolerability and complications after robotic-assisted transperineal MRI-US-fusion guided biopsy of the prostate (RA-TP-PB_x_). An upcoming PB_x_ bearing uncertainty regarding a suspected malignant disease as well as the interventional risks, poses a physical and psychological burden for patients. Therefore, the ideal biopsy technique is as painless as possible and combines low complication rates with upmost diagnostic precision. The aim of this study was to evaluate the MonaLisa prostate biopsy system in terms of safety, tolerability, and patient-related outcomes.

## Materials and methods

This prospective study analyses the safety profile and functional results of 228 patients, who had undergone RA-TP-PB_x_ at the University Hospital Basel between January 2020 and June 2022. Indication for biopsy resulted from suspicious DRE, elevated PSA values or suspicious lesions in mpMRI. Imaging was performed in all patients prior to biopsy, suspicious lesions were classified according to PI-RADS v2.1. The study was approved by the local ethics committee (ID 2020-01381) and was performed in accordance with the Declaration of Helsinki. All patients provided written informed consent. Side effects, clinical, functional, histological, and demographic data were collected and assessed. In addition, medication for male urinary dysfunction, type of anticoagulation and immunodeficiency, including diabetes mellitus type 2, immunosuppressants or acquired immune deficiency syndrome (AIDS), were recorded.

### Biopsy technique

**Figure 1:**
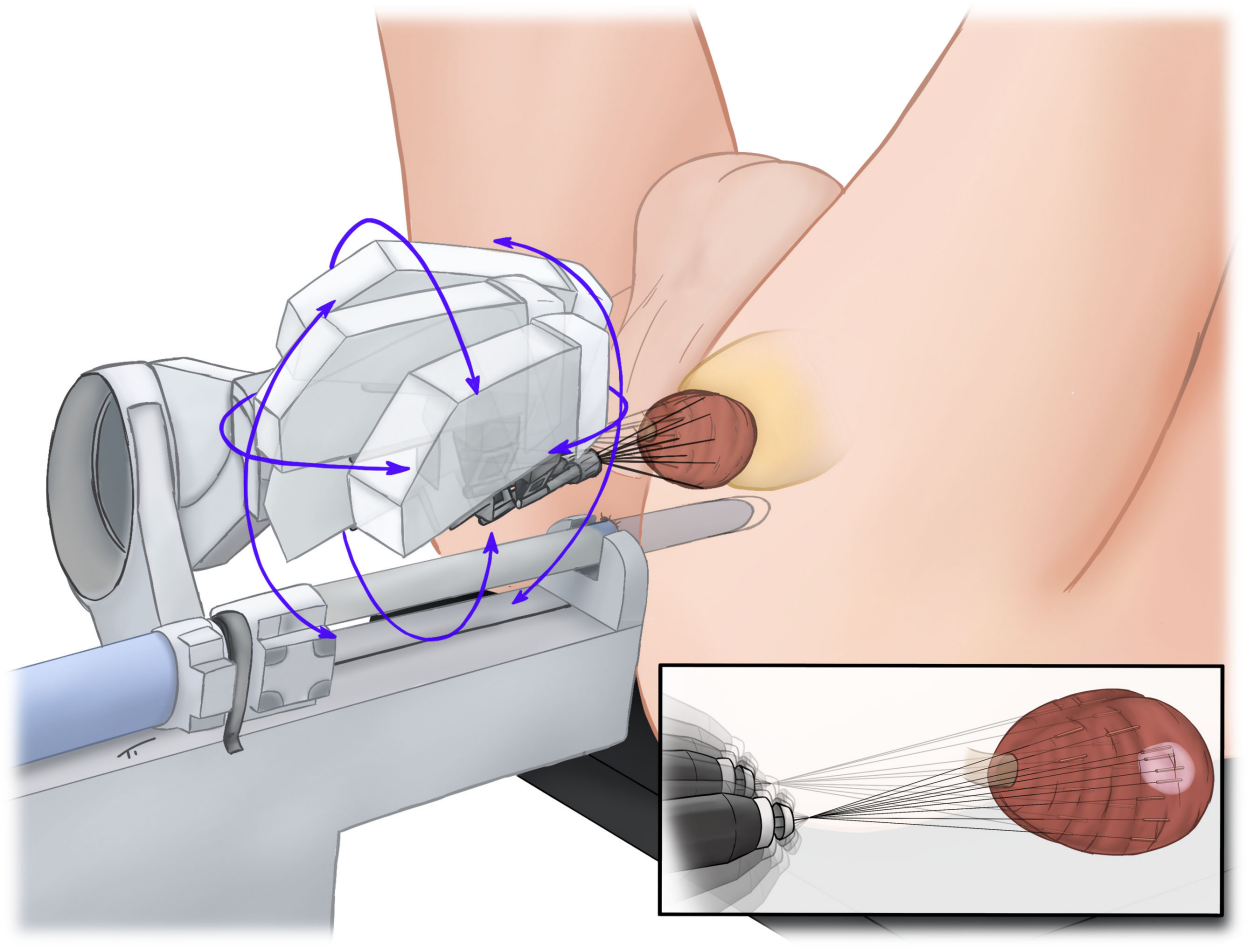
Robotic-assisted transperineal MRI-TRUS-fusion guided biopsy of the prostate.

A 3D model of the prostate, including suspicious lesions, was generated by a skilled team of radiologists (DJW, PB) and RA-TP-PBx was performed with an iSR’obot™ MonaLisa device (Biobot^©^) (**Figure 1**) by one experienced surgeon (CW). Anticoagulation with factor Xa inhibitors and phenprocoumon was discontinued and bridged with low-molecular-weight heparin according to the individual risk of a thromboembolic event. Therapy with acetylsalicylic acid was continued and was used to bridge patients under therapy with clopidogrel. Standardized, anti-infective prophylaxis was administered to the first 60 (26.3%) patients. After the initial implementation phase of the new biopsy technique anti-infective prophylaxis was omitted if not indicated by positive findings in preoperative urine culture. After RA-TP-PB_x_ no transurethral catheter was used by default. A detailed description of our procedure has already been published previously (9).

### Analysis and statistical methods

Validated questionnaires, including “International Prostate Symptom Score” (IPSS) with quality of life (QoL), “International Consultation on Incontinence Questionnaire – Urinary Incontinence” (ICIQ), and “National Institutes of Health - Chronic Prostatitis Symptom Index” (NIH-CPSI) were used to assess functional outcome before and about one week after biopsy. Additionally, the occurrence of side-effects including acute urinary retention (AUR), gross hematuria, hematospermia, pain according to visual analog scale for pain (VAS, 1 – 10 points), urinary tract infections (UTI), local complications and patient satisfaction were collected and analyzed.

Database was created using Excel (Microsoft^©^), statistical analyses were performed with SPSS Statistics 24.0 (IBM^©^). The Chi-squared and Fisher`s exact tests were used to compare nominal data. For determination of significant differences among the normally distributed data the Student`s t test (dependent/independent) was applied. Logistic regression was used for binary classification, i.e. to estimate the posterior probability of a binary response based on a list of independent predictor variables. This probability is described by a generalized linear model. Odd`s ratio was performed for risk assessment. All tests were performed at a two-sided significance level of α = 0.05.

## Results

Transperineal robotic-assisted biopsy of the prostate was successfully performed in 228 men with suspicious mpMRI-lesions and/or PSA-constellation. Mean (range) age, PSA, and prostate volume (PV) were 64.9 years (46 – 84), 11.8 ng/ml (0.2 - 561) and 48.4 ml (9 – 310), respectively. Detailed patient baseline characteristics are summarized in **Table 1**. At the time of biopsy, 63/228 (27.6%) patients took regular medication for male urinary dysfunction. 59/228 (25.9%) took anticoagulant medication, of which 38 patients had biopsy under ongoing antiplatelet therapy. 38/228 (16.7%) patients presented a form of immunodeficiency as stated in the methods. Mean pain score on the day of biopsy was 1.3 points on VAS, which remained constant on the day after biopsy (1.2 points). Overall, 32 of 228 patients (14%) developed grade I complications according to Clavien-Dindo classification. No higher-grade complications occurred. The most common side-effect observed after biopsy was gross haematuria 145/228 (63.6%), which was self-limiting and none of these patients required treatment. 59/145 (25.9%) patients reported gross haematuria duration of more than 3 days. Hematospermia occurred in 98/228 (43%) patients. Anticoagulant therapy, continued antiplatelet medication, PV (≥ 40 ml), biopsy proven inflammation or number of biopsy cores (≥ 25) had no significant influence on occurrence of haematuria/-spermia. Acute urinary retention (AUR) occurred in 32/228 (14%) patients. Patients who developed AUR had a significant higher baseline IPSS-Score (13.2 vs. 10; p 0.02), a bigger prostate volume (61.4 vs. 46 ml; p 0.008) and more biopsy cores taken (29 vs. 25; p 0.009). However, number of biopsy cores (≥ 25), PV (≥ 40 ml) and medication for male urinary dysfunction couldn`t be identified as individual risk factors for the occurrence of AUR. IPSS ≥ 8 (moderate/severe symptoms) and biopsy proven inflammation showed only a tendential association to an increased risk of AUR (OR = 2.49 and 2.29, respectively). Using multivariate multiple regression, only for AUR a significant overall model (p = 0.04) was demonstrated, with none of the predictors providing a clear prediction. Significant influence was shown for IPSS ≥ 8 on “Change of IPSS”, although this result is considered random with regard to the insignificant overall model. No statistically significant change of functional scores (IPSS, QoL and ICIQ) occurred in our cohort shortly after biopsy. One patient (0.4%) developed urinary tract infection (UTI). 66/228 (28.9%) had undergone prostate biopsy priorly. 48/66 (84.2%) of these patients favored transperineal robotic-assisted biopsy over all other methods and rated transperineal robotic-assisted biopsy as the most pleasant biopsy approach. Regarding local conditions, haematoma at puncture, local skin infection and bleeding from puncture site occurred in 8/228 (3.5%), 0/228 (0%) and 10/228 (4.4%), respectively. Detailed data for functional outcome and side-effects are summarized in **Table 2**. Notably, no patients with immunodeficiency developed any infectious complications.

**Table 1:** Baseline characteristics.

Parameter	Patients (n)	Mean ± SD (range)
Age (years)	228	64.9 ± 7.6 (46 – 84)
Prostate volume (cm^3^)	228	48.4 ± 30.1 (9 – 310)
Serum PSA (ng/ml)	226	11.8 ± 39.1 (0.2 – 561)
Number of biopsy cores (total)	228	25.6 ± 8.2 (5 – 51)
IPSS	216	10.5 ± 7.2 (0 – 34)
ICIQ	210	1.2 ± 2.6 (0 – 14)
QoL	217	1.6 ± 1.5 (0 – 5)
NIH-CPSI (total)	173	7.8 ± 6.4 (0 – 40)
NIH-CPSI (pain)	173	1.7 ± 3 (0 – 21)
NIH-CPSI (micturition)	173	2.8 ± 2.2 (0 – 10)
NIH-CPSI (Quality of life)	173	3.5 ± 2.9 (0 – 12)
	**Patients (n)**	**%**
Suspicious DRE	36	15.8
Under “Active surveillance”	31	13.6
Previous biopsy	66	28.9
Immunodeficiency	38	16.7
Medication for MUD	63	27.6
Anticoagulation	59	25.9
Perioperative antibiotic prophylaxis/therapy	76	33.3

SD, standard deviation; PSA, prostate-specific antigen; IPSS, international prostate symptom score; ICIQ, international consultation on incontinence questionnaire; QoL, quality of life; NIH-CPSI, chronic prostatitis symptom index; DRE, digital rectal examination; MUD, male urinary dysfunction.

**Table 2:** Functional outcomes and side effects.

Parameter	Mean ± SD (range)	Mean ± SD (range)	p – value^*^
	Before biopsy	After biopsy	
IPSS	10.5 ± 7.2 (0 – 34)	11 ± 7.4 (1 – 34)	0.23
ICIQ	1.2 ± 2.6 (0 – 14)	1.6 ± 2.8 (0 – 13)	0.12
QoL	1.6 ± 1.5 (0 – 5)	1.7 ± 1.5 (0 – 5)	0.44
**Parameter**	**Mean ± SD (range)**		**p – value^#^**
Pain on the day of biopsy	1.3 ± 1.9 (0 – 9)		0.68
Pain on following day	1.2 ± 1.9 (0 – 10)		
Change of IPSS^1^	0.4 ± 5.2 [(-) 31 – (+) 22]		–
Change of ICIQ^1^	0.3 ± 2.3 [(-) 13 – (+) 8]		–
Change of QoL^1^	0.1 ± 1.4 [(-) 5 – (+) 5]		–
**Parameter**	**Total (n)**		**%**
Acute urinary retention	32		14
Gross hematuria	145		63.6
Duration of hematuria (1 day)	34		14.9
Duration of hematuria (2-3 days)	51		22.4
Duration of hematuria (>3 days)	59		25.9
Hematospermia	98		43
Urinary tract Infection	1		0.4
Perineal bleeding	10		4.4
Perineal hematoma	8		3.5
Skin infection	0		0
Histology-proven Inflammation	73		32
Negative biopsy	95		40
Positive biopsy	133		60

SD, standard deviation; IPSS, international prostate symptom score; ICIQ, international consultation on incontinence questionnaire; QoL, quality of life.

^1^change of functional parameters (-) decrease of score after biopsy, (+) increase of score after biopsy.

^*^ p – value determined by a dependent Student`s t test.

**^#^** p – value determined by an independent Student`s t test.

Sub-group-analysis for the functional outcome and side effects and subgroup specifications are summarized in **Table 3** **Supplementary Table 1**, respectively.

**Table 3:** Functional outcome and side-effects - Subgroup analysis.

Parameter	MMUDyes/no	ACyes/no	BC<25/≥25	INFyes/no	PV<40/≥40	IPSS<8/≥8	MMR
	p – value^*^						
Acute urinary retention	0.63	0.19	0.33	0.06	0.57	0.06	0.04
Gross hematuria	0.7	0.97	0.45	0.3	0.69	0.3	0.75
Hematospermia	0.5	0.17	0.45	0.45	0.55	0.03	0.17
Perineal hematoma	0.18	0.55	0.59	0.45	0.97	0.92	0.71
Change of IPSS**^1^**	0.55	0.75	0.72	0.28	0.52	0.01	0.09
Change of ICIQ**^1^**	0.73	0.58	0.32	0.85	0.75	0.19	0.75
**Parameter**	**Complication**		**n**	**Mean ± SD (range)**			**p –value^#^**
PV	AUR no		192	46 ± 24.1 (9 – 217)			0.009
	AUR yes		31	61.4 ± 52.2 (27.5 – 310)			
Number of biopsy cores	AUR no		196	25 ± 7.9 (5 – 46)			0.009
	AUR yes		32	29 ± 9.2 (5 – 51)		
IPSS (before biopsy)	AUR no		185	10 ± 7.1 (0 – 32)			0.02
	AUR yes		30	13.2 ± 7.6 (3 – 34)		
PV - Pain on day of biopsy	No pain		103	44.6 ± 17.8 (18 – 88)			0.4
	> 0 points		107	47.1 ± 24.5 (9 – 173)		
PV - Pain on following day	No pain		111	45.5 ± 20.3 (9 – 120)			0.8
	> 0 points		99	46.4 ± 22.9 (14 – 173)		
BC - Pain on day of biopsy	No pain		104	26.6 ± 7.9 (5 – 51)			0.25
	> 0 points		108	24.5 ± 8.4 (5 – 41)		
BC - Pain on following day	No pain		112	26.3 ± 8.1 (5 – 51)			0.56
	> 0 points		100	24.7 ± 8.3 (6 – 41)		

NIH-CPSI, chronic prostatitis symptom index; INF, histology-proven inflammation; MMUD, medication for male urinary dysfunction; AC, anticoagulation; BC, biopsy cores; PV, prostate volume; IPSS, international prostate symptom score; MMR, multivariate multiple regression (overall model); ICIQ, international consultation on incontinence questionnaire; SD, standard deviation; AUR, acute urinary retention.

^*^ p – value determined using multivariate multiple regression.

**^#^** p – value determined by an independent Student`s t test.

^1^change of functional parameters (-) decrease of score after biopsy, (+) increase of score after biopsy.

## Discussion

To the best of our knowledge, this is the first prospective study to evaluate safety, tolerability, side effects, and functional outcome of transperineal robotic-assisted prostate biopsy. Transrectal ultrasound-guided biopsy of the prostate still is used as the standard approach for obtaining representative samples for identification and classification of PCa (10). However, the current EAU Guidelines 2022 clearly favor the perineal access route, due to the lower risk of infectious complications (1). Our study reports the outcomes of robotic-assisted perineal biopsy, that requires only two puncture sites. The applied sampling strategy provides histologic evaluation of the entire gland including suspicious lesions (9). Overall, 14% of our patients developed grade I complications according to Clavien-Dindo classification. The superior tolerability of the RA-TP-PB_x_ is highlighted by the mean value of 1.3 points on VAS for pain on the day of and 1.2 points on the days after biopsy. TP-PB_x_ performed under general anesthesia also displays a favorable pain profile (VAS 1.3) as compared to TP-PB_x_ (VAS 2) and TR-PB_x_ (VAS 2) in local anesthesia (11). Furthermore, most patients (84.2%) of our cohort having undergone conventional non-robotic biopsy, preferred RA-TP-PB_x_. Although, feasibility of the TP-PBx in local anesthesia was shown in various studies (6, 12), general anesthesia is recommended in RA-TP-PB_x_ in order to enable maximum diagnostic accuracy. Hematuria and hematospermia were identified as most common side effects. Rates of occurrence were comparable to other studies reporting sides effects of TP-PB_x_ and TR-PB_x_ (13). Notably, none of our patients developed significant gross hematuria requiring bladder irrigation. A further advantage of the TP-PB_x_ is the absence of hematochezia or rectal bleeding, which is described with an incidence of up to 45% in transrectal biopsy (3). In our cohort, the rate of AUR after RA-TP-PB_x_ was 14%, which is comparable to the study of Pepe et al. with 11.1% on saturation TP-PB_x_ and > 24 cores taken (14), yet higher than in studies with lower number of biopsy cores taken (10–18) with rates of AUR ranging from 1.4% to 6.7% (15, 16). Even though the number of biopsy cores is considered a risk factor for AUR (14), the number of cores (≥25) had no significant impact on the risk of appearance of an AUR in our cohort applying a target saturation approach (9). Using multivariate multiple regression, an significant overall model (p = 0.04) for AUR was shown, with none of the predictors providing a clear prediction. RA-TP-PB_x_ allows for complete diagnostic coverage of the prostate *via* only two puncture sites. This sterile and minimally invasive approach resulted in the occurrence of only one UTI (0.4%) requiring intravenous antibiotic treatment. Notably, this patient had received antibiotic treatment with oral cephalosporine according to resistency profile, however the duration of pre-treatment (single dose) turned out to be insufficient given the histopathology also revealed acute inflammation. The rate of UTI is comparable to other studies reporting rates of UTI after TP-PBx between 0 – 0.7% (15–17). In contrast, TR- PB_x_ is associated with higher rates of infectious complications ranging between 2 - 5% despite antibiotic prophylaxis (11, 18, 19). In line with the study of Günzel et al. (11), omission of standard perioperative antibiotic prophylaxis in TP- PB_x_ did not result in a significant increase of infections. Notably, none of the immunodeficient patients developed infectious complications indicating that the sterile and minimally invasive biopsy technique enables to safely omit perioperative antibiotic prophylaxis even in patients at special risk for the development of infectious complications. Requiring no antibiotic prophylaxis helps to reduce the risk of antibiotic related complications and the development of drug resistant bacteria. Our results corroborate the findings from other groups (20). However, single center data, limited patient number and non-randomized trial design without a control group represent limitations of this study. Further studies are required to confirm our results. Nevertheless, this work indicates the superior safety profile of robotic assisted transperineal prostate biopsy as compared to a transrectal approach. We assume that the minimally invasive biopsy technique *via* only two entry points diminished local tissue trauma and subsequently reduced the risk for infectious complications.

## Data availability statement

The raw data supporting the conclusions of this article will be made available by the authors, without undue reservation.

## Ethics statement

The studies involving human participants were reviewed and approved by Ethikkommission Nordwest- und Zentralschweiz. The patients/participants provided their written informed consent to participate in this study.

## Author contributions

All authors have conjointly designed the study, and MW, PT, and CW interpreted the data and drafted the manuscript. AM supported data collection and patient care. All authors designed and critically revised the manuscript for important intellectual content. MW, PT, and CW were involved in the statistical analysis. All authors contributed to the article and approved the submitted version.

## Conflict of interest

Author CW was supported by grants from Siemens Healthineers and Uromed.

The remaining authors declare that the research was conducted in the absence of any commercial or financial relationships that could be construed as a potential conflict of interest.

## Publisher’s note

All claims expressed in this article are solely those of the authors and do not necessarily represent those of their affiliated organizations, or those of the publisher, the editors and the reviewers. Any product that may be evaluated in this article, or claim that may be made by its manufacturer, is not guaranteed or endorsed by the publisher.

## Supplementary material

Supplementary Table 1 Subgroups specifications.

## Abbreviations

5-ARI, 5-alpha-reductase inhibitor; AC, anticoagulation; AIDS, acquired immune deficiency syndrome; AUR, acute urinary retention; BC, biopsy cores; DRE, digital rectal examination; ICIQ, International Consultation on Incontinence Questionnaire – Urinary Incontinence; IPSS, International Prostate Symptom Score; i.v., intravenous; INF, histology-proven inflammation; LUTS, lower urinary tract symptoms; mpMRI, multiparametric MRI; MRI, magnetic resonance image; MUD, male urinary disfunction; MMUD, medication for male urinary dysfunction; NIH-CPSI, National Institutes of Health - Chronic Prostatitis Symptom Index; PB_x_, biopsy of the prostate; PCa, Prostate cancer; p.o., per os; PSA, prostate specific antigen; PV, prostate volume; QoL, quality of life; RA-TP-PB_x_, Robotic-assisted transperineal MRI-US-fusion guided biopsy of the prostate; SD, standard deviation; TP, transperineal; TP-PB_x_, transperineal biopsy of the prostate; TR, transrectal; TR-PB_x_, transrectal biopsy of the prostate; TRUS, transrectal ultrasound; UTI, urinary tract infections; VAS, visual analog scale.
