# Supplementary material for: Safety profile of robotic-assisted transperineal MRI-US-fusion guided biopsy of the prostate
Source: Front Oncol. 2022 Dec 1;12:1025355. doi: 10.3389/fonc.2022.1025355 (PMC9751759; doi:10.3389/fonc.2022.1025355)
Supplement: Supplementary Table 1 — Subgroups specifications. [file Table_1.docx]

**Supplementary Table 1** Subgroups specifications

| Parameter | Total cohort of 228 patients | |
| --- | --- | --- |
| Anticoagulation | n | % |
| Total | 59 | 25.9 |
| Acetylsalicylic acid | 32 | 14 |
| Clopidogrel | 6 | 2.2 |
| Factor Xa inhibitors | 16 | 6.6 |
| Phenprocoumon | 5 | 2.2 |
| Medication for male urinary dysfunction | n | % |
| Total | 63 | 27.6 |
| Alpha-blocker monotherapy | 40 | 17.5 |
| Alpha-blocker + 5-ARI | 7 | 3.1 |
| 5-ARI | 3 | 1.3 |
| Tadalafil | 2 | 0.9 |
| Phytotherapy | 1 | 4.8 |
| Immunodeficiency | n | % |
| Total | 38 | 16.3 |
| Diabetes mellitus type 2 | 19 | 8.3 |
| Immunosuppressants | 15 | 6.6 |
| AIDS | 4 | 1.8 |
| Antibiotic prophylaxis | n | % |
| Total | 76 | 33.3 |
| Cefuroxim i.v. | 61 | 26.8 |
| Third-generation cephalosporin i.v. | 3 | 1.3 |
| Aminopenicilline p.o. | 4 | 1.8 |
| Co-trimoxazol p.o. | 6 | 2.6 |
| Ciprofloxacin p.o. | 1 | 0.4 |
| Cefpodoxim p.o. | 1 | 0.4 |

*5-ARI*, 5-alpha-reductase inhibitor; *AIDS*, acquired immune deficiency syndrome; *i.v.*, intravenous; *p.o.*, per os
